# Ketogenic effects of milk protein–coated medium-chain triglycerides in healthy men: A randomized, double-blind, crossover study

**DOI:** 10.1371/journal.pone.0355475

**Published:** 2026-08-10

**Authors:** Kentaro Ito, Yuichi Kawasaki, Kazuji Tamura, Kentaro Nakamura, Yoshitaka Kawai, Tadashi Watanabe

**Affiliations:** 1 R&D Division, Meiji Co., Ltd, Tokyo, Japan; 2 Watanabe Hospital, Tokyo, Japan; Oita University Faculty of Medicine, JAPAN

## Abstract

Medium-chain triglycerides (MCTs) have ketogenic effects and are reportedly effective in improving cognitive and physical functions. We hypothesized that coating the MCT microparticles with protein can enhance their absorption in the intestinal tract and their ketogenic effects. This randomized, double-blind, crossover study examined the ketogenic effects of a single intake of milk protein–coated MCT (coated MCT) in healthy adults. Participants ingested 6 g of coated MCT or untreated MCT. Then, we measured the blood levels of ketone bodies and medium-chain fatty acids and gastrointestinal symptoms up to 6 h later. The coating treatment did not significantly affect the area under the curve of change in blood total ketone bodies (TKB) (primary outcome) (*P* > 0.05). Meanwhile, the maximum concentration of changes in TKB, β-hydroxybutyrate, acetoacetate, and octanoic acid (secondary outcome) was significantly higher in coated MCT than in untreated MCT (*P* < 0.05). Furthermore, TKB positively correlated with octanoic acid (*P* < 0.05). Both coated and untreated MCT showed mild gastrointestinal symptoms, with no significant difference observed between the groups (*P* > 0.05). Coated MCT may enhance the ketogenic effect and can be safely consumed. These findings are expected to support the development of new ketogenic therapeutic strategies.

## Introduction

Medium-chain triglycerides (MCTs) are fats composed of medium-chain fatty acids (MCFAs). MCFAs generally refer to fatty acids with 8–12 carbon atoms. They are found in foods such as coconut oil and cow’s milk. MCT has ketogenic effects [1–4]. Ingested MCT is degraded by lipase more rapidly than long-chain triglycerides (LCT). Unlike long-chain fatty acids (LCFAs), MCFAs have been reported to be transported to the liver via the portal vein, at least in part [5, 6]. In the liver, MCFAs are β-oxidized faster than LCFAs [7]. Some of the resulting acetyl-CoAs are converted into ketone bodies. Ketone bodies, which include acetoacetate and β-hydroxybutyrate, are used for energy not only in tissues such as skeletal muscle but also in the brain [8, 9]. Ketone bodies have been reported to play various physiological roles. For example, studies in mice have reported that ketone bodies, particularly β-hydroxybutyrate, are involved in feeding regulation [10, 11]. Furthermore, octanoic acid, a type of MCFA, activates ghrelin, an appetite-promoting and anabolic peptide hormone, by binding to it. Some studies have reported that continuous intake of MCT can increase activated ghrelin in humans [12–14]. MCT intake is reportedly effective in improving cognitive function [15–18] and muscle strength [19–21] among older adults.

High MCT intake causes gastrointestinal symptoms such as stomachache, indigestion, and diarrhea [15,22–24]. This may be solved by enhancing the ketogenic effect of MCTs even in lower doses. For example, emulsifying MCT can enhance the ketogenic effect [22]. Another solution is to mitigate gastrointestinal symptoms caused by MCT ingestion. Gastric discomfort may also be related to the production of free MCFAs by lipase degradation in the stomach. Both MCT and LCT are degraded by lingual lipase, but MCT is degraded 5–8 times faster than LCT and is also degraded in the stomach [25]. Therefore, gastric mucosa irritation by free MCFAs is one of the factors of gastrointestinal symptoms. Hence, a method utilizing MCT with higher ketogenic effects and fewer side effects should be developed.

We hypothesized that coating the microparticulated MCTs with protein allows them to reach the small intestine while maintaining their particulate state, thereby enhancing its absorption in the intestine and its ketogenic effects and reducing gastric discomfort by inhibiting hydrolysis in the stomach. On the basis of this hypothesis, we developed MCTs coated with milk protein (coated MCT). In this study, we aimed to evaluate the effect of coating MCT with milk protein on the ketogenic effect and gastrointestinal symptoms. We compared the ketogenic effect of coated MCT with untreated MCT in a randomized, double-blind, two-period, crossover study in healthy adults.

## Materials and methods

### Preparation of coated MCT

In the preparation, 7.7% (w/w) milk protein (whey protein isolate and sodium caseinate) and 18.2% (w/w) MCT oil (octanoic acid:decanoic acid ratio, 75:25; Nisshin Oillio, Tokyo, Japan) were dissolved or dispersed in hot water (45 °C–55 °C). A homomixer (Homo mixer, PRIMIX Corp., Hyogo, Japan) was used for pre-emulsification at 8000 rpm for 20 min and a high-pressure homogenizer (TwinPanda, GEA Inc., Germany) for homogenization (30 MPa + 5 MPa). Next, the mixture was heated at 105 °C for 15 s, and spray-dried using a spray dryer (MOBILE MINOR, GEA Inc., Germany) under the following conditions: inlet air temperature, 185 °C; outlet air temperature, 72 °C–78 °C, and liquid feed rate, 2 L/h.

The structure of the coated MCT was observed using a confocal microscope. Lipids and proteins were stained with BODIPY 493/503 and Nile Blue A, respectively and images were then taken using a confocal microscope (FV1000, Olympus Corp., Tokyo, Japan). These images confirmed that most of the oil droplets were coated with proteins (S3 Fig).

### Clinical trial design

The clinical trial was a double-blind, randomized, two-arm, two-period crossover design. Participants were randomly assigned to coated MCT in period 1 followed by untreated MCT in period 2 (C to U sequence), or untreated MCT in treatment period 1 followed by coated MCT in period 2 (U to C sequence). Each period was separated by a 6-day washout. We chose a crossover design because the within-subject variation is less than the between-subject variation, thereby requiring fewer participants.

### Ethics statement

This study conformed to the Declaration of Helsinki and Ethical Guidelines for Medical and Health Research Involving Human Subjects published by Japan’s Ministry of Education, Culture, Sports, Science and Technology and the Ministry of Health, Labor, and Welfare. Additionally, it was approved by the Ethics Review Committee of Watanabe Hospital (Review number: FA6–2309_F05_001_000_20231220_1, Date of approval: December 20, 2023). The study protocol was registered at UMIN-CTR (ID: UMIN000053245, Registered date: December 27, 2023, https://rctportal.mhlw.go.jp/en/detail?trial_id=UMIN000053245) before the start of the study. We conducted this study at Watanabe Hospital (Tokyo, Japan). The recruitment was started on January 15, 2024, and the last follow-up was ended on March 31, 2024.

### Study participants

The inclusion criteria were as follows: 1) provision of a written informed consent form with the participant’s signature, 2) a male aged 20–40 years, and 3) a body mass index of 18.5–25.0 kg/m^2^. The exclusion criteria were as follows: 1) consumed drugs, supplements, functional foods, MCT, or coconut oil at least 2 days a week within 1 month before the screening test (SCR); 2) had serious chronic diseases; 3) had abnormalities in lipid metabolism, glucose metabolism, liver function, or renal function; 4) had food allergies; 5) had lactose intolerance; 6) had gastrointestinal surgery history; 7) smoking habit; 8) donated 200 mL of blood within 1 month before SCR or 400 mL of blood within 3 months before SCR; 9) participated in other clinical studies within 1 month before SCR or planned to participate in other clinical studies within the study period; and 10) deemed inappropriate by the principal investigator.

Given the lack of previous studies on coated MCT or similar materials, the number of participants was set at 12 according to a previous study on the ketogenic effect of MCT [2, 22].

### Test foods

Powdered foods containing 6 g of untreated MCT (dextrin-coated MCT powder, Nisshin Oillio, Tokyo, Japan) or coated MCT were prepared (Table 1). We also prepared a powdered food containing 6 g of LCT. The LCT food was used in the SCR2 and compared with each MCT food as a secondary analysis. All test foods were packaged in plain aluminum pouches so that they could not be identified by the researchers and the participants. All foods were manufactured by Meiji Co., Ltd. (Tokyo, Japan). The test food representative coded and blinded the test foods and prepared an identification table, which was sealed and sent to the allocation manager (Statcom Company, Tokyo, Japan).

**Table 1:** Nutritional contents of the test foods (per day).

	Coated MCT foods	Untreated MCT foods	LCT foods
**Energy (kcal)**	98.4	98.5	98.5
**Proteins (g)**	6.6	6.6	6.6
**Lipids (g)**	6.1	6.1	6.1
**MCT (g)**	6.0	6.0	0.0
**LCT (g)**	0.1	0.1	6.1
Octanoic acid (g)^*^	4.1	4.1	0.0
Decanoic acid (g)^*^	1.4	1.3	0.0
**Carbohydrates (g)**	4.5	4.5	4.5

* Octanoic acid and decanoic acid were determined by gas chromatography. MCT, medium-chain triglycerides; LCT, long-chain triglycerides.

### Study flow

The principal investigator explained the study thoroughly to potential participants and obtained their written informed consent. The eligible participants underwent two SCRs. In SCR1, participants ingested untreated MCT food and blood was collected before and 1.5 h after ingestion. We selected 18 participants from those closest to the median in blood total ketone bodies (TKBs). In SCR2, the participants ingested LCT food; blood was collected over time and blood parameters were measured according to the MCT ingestion test in period 1 and period 2 tests. We selected 12 participants from those with low ΔAUC (defined in the Statistical Analysis subsection) of blood TKB after LCT intake. The LCT results were then compared with the MCT results as the secondary analysis.

The allocation manager allocated 12 subjects to the two groups in a 1:1 ratio using a stratified permutation block method, with age and blood TKBs at SCR1 used as indicators. The allocation manager also prepared an allocation table and locked it with the sealed identification table to keep the participants and researchers blinded until key opening.

After allocation, the period 1 and period 2 tests were conducted with a washout period of at least 6 days. The participants stayed at the clinic from the day before each test date, and they were prohibited from drinking alcohol during this period. They were also prohibited from eating or drinking anything except water after 21:00 on the day before the test. On the test day, they were instructed to consume a standard meal (a jelly drink containing 45 g carbohydrates) as breakfast and were prohibited from eating or drinking anything other than water until the end of the test.

Furthermore, we instructed them to ingest the coated MCT food or untreated MCT food with 200 mL of water within 2 min. Venous blood was collected before ingestion and 0.5, 1, 2, 3, 4, 5, and 6 h after ingestion. At each time, they completed a gastrointestinal symptom questionnaire. A physician interviewed them before and 6 h after ingestion of the test food. Their physical condition was confirmed via telephone call or e-mail the following day or later.

### Blood metabolites and markers

Plasma samples were prepared from the collected blood, using conventional methods. Plasma MCFAs were measured using gas chromatography–mass spectrometry (7890B, 7000D; Agilent Inc., Santa Clara, CA) with ZB-FAME column (30 m × 0.25 mm × 0.2 µm, Phenomenex Inc., Torrance, CA) after methylesterification using a kit (Nacalai Tesque, Inc., Kyoto, Japan). Gas chromatography was conducted using a split injection mode (split ratio 1:10) with helium as the carrier gas at a flow rate of 1.2 mL/min. The oven temperature program was as follows: initial temperature of 70 °C held for 2 min, increased at 10 °C/min to 200 °C, then at 3 °C/min to 230 °C, and finally at 30 °C/min to 260 °C, held for 2 min. Mass spectrometry was performed in selected ion monitoring mode. The interface and ion source temperatures were set to 260 °C and 200 °C, respectively. Retention times and m/z values of each compound were as follows: octanoic acid, retention time 4.9 min, ions 74, 87, 127 m/z and decanoic acid, retention time 7.0 min, ions 74, 87, 101 m/z. Active ghrelin and desacyl ghrelin in plasma were measured using an enzyme-linked immunosorbent assay kit (Bertin Technologies SAS, Montigny-le-Bretonneux, France) according to the manufacturer’s protocol. For other items, blood samples were assayed by LSI Medience Co., Ltd. (Tokyo, Japan). β-Hydroxybutyrate (BHB) and acetoacetate (AcAc) in the blood were measured via enzymatic method. TKB was defined as the sum of BHB and AcAc in the blood. Active ghrelin and desacyl ghrelin were measured only before and 2, 4, and 6 h after ingestion.

### Gastrointestinal symptoms questionnaire

The participants rated their gastrointestinal symptoms (stomachache, indigestion, nausea, bloating, and fullness) on a 3-point scale of 0 (no symptoms), 1 (symptoms recognized but tolerable), and 2 (symptoms of unacceptable intensity).

### Adverse events

In this study, adverse events were defined as those that newly occurred from the day before the intake of the test foods to the day after the period 2 test. However, if the event was observed before the start of the study and did not worsen after the start of the study, or if the physician made a medical judgment that the occurrence of the event was expected based on the condition of the subject before the start of the study, the event was not treated as an adverse event.

### Statistical analysis

The data are presented as means and standard deviations. The change between the pre- and post-intake at each time point (Δvalue), the area under the curve (AUC), the AUC of Δvalue (ΔAUC), the maximum concentration (*C*_max_), the *C*_max_ of Δvalue (Δ*C*_max_), and the time to reach *C*_max_ (*T*_max_) were calculated. The AUC was calculated using the trapezoidal method. The test foods were compared using Wilcoxon’s signed-rank test. We also employed Wilcoxon’s signed-rank test (Bonferroni correction) for comparing pre- and post-intake at each time point and for the secondary analysis wherein each MCT diet was compared with the LCT diet. Regarding safety, the number of adverse events was tabulated, and the presence of adverse events was compared between groups using McNemar’s test. Differences were considered significant when the *P*-value was less than 0.05. Missing data were not imputed. All statistical data were analyzed using BellCurve for Excel (Social Survey Research Information Co., Ltd., Tokyo, Japan).

The efficacy analysis set was the per-protocol set. The safety analysis set included all participants who ingested the test food at least once.

The primary outcome was the ΔAUC of TKB. The secondary outcomes were the AUC, ΔAUC, *C*_max_, Δ*C*_max_, and *T*_max_ in metabolite markers and gastrointestinal symptoms.

## Results

### Participants

**Fig 1:**
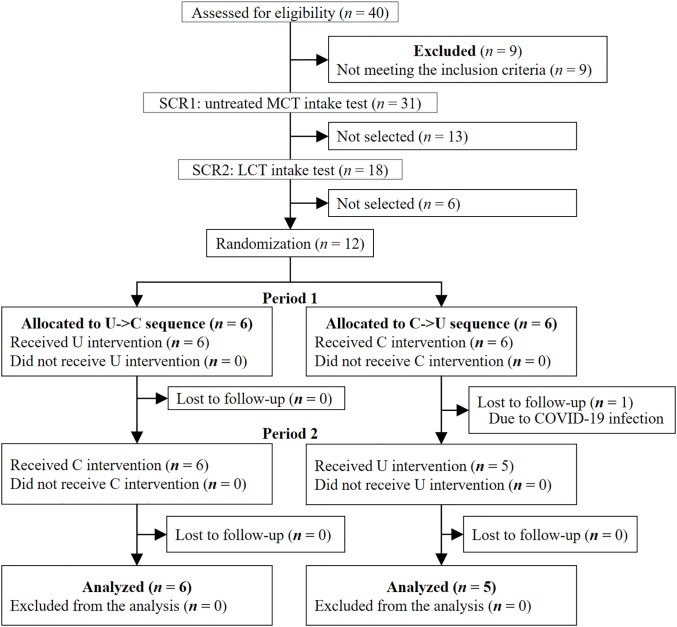
Participant flow. U, untreated MCT; C, coated MCT; MCT, medium-chain triglycerides; LCT, long-chain triglycerides; SCR, screening test.

Out of 40 adult males who participated, 9 were excluded after eligibility assessment because of high blood test values (*n* = 6), HIV positivity (*n* = 1) and COVID-19 positivity (*n* = 2). Twelve participants were selected from the results in SCR1 and SCR2. One participant in the C to U sequence was withdrawn before period 2 because of COVID-19 infection. As a result, 6 in the U to C sequence and 5 in the C to U sequence were analyzed (Fig 1).

Table 2 shows the baseline demographic characteristics of the participants. None of the characteristics significantly differed between the sequences. In addition, no abnormalities were observed in the blood test results of the participants (S6 Table).

**Table 2:** Baseline demographic characteristics.

		U to C sequence (*n* = 6)	C to U sequence (*n* = 5)	*P*-value
**Age**	Years	29.7 ± 8.5	30.0 ± 7.9	1.000
**Height**	cm	170.7 ± 10.6	168.1 ± 1.9	0.689
**Body weight**	kg	63.3 ± 9.6	66.4 ± 5.2	0.575
**BMI**	kg/m^2^	21.6 ± 1.4	23.5 ± 1.6	0.065

Values are presented as means ± standard deviation. Sequences were compared using the Wilcoxon rank sum test. U, untreated MCT; C, coated MCT; MCT, medium-chain triglycerides; BMI, body mass index.

### Primary outcome

The ΔAUC of TKB was 1038 ± 468 μmol/L·h for coated MCT and 937 ± 385 μmol/L·h for untreated MCT, showing no significant difference (*P* = 0.47; Table 3).

**Table 3:** Pharmacokinetic parameters of total ketone bodies.

	Unit	untreated MCT	coated MCT	LCT
**AUC**_**0-6h**_	μmol/L·h	1070 ± 384	1298 ± 618	762 ± 444
**ΔAUC**_**0-6h**_	μmol/L·h	937 ± 385	1038 ± 468	669 ± 431
***C***_**max**_	μmol/L	329 ± 118	446 ± 129*^#^	243 ± 151
**Δ*C***_**max**_	μmol/L	307 ± 118	424 ± 130*^#^	227 ± 149
***T***_**max**_	h	2.4 ± 1.6^#^	1.2 ± 1.0*^#^	4.3 ± 1.0

Values are presented as means ± standard deviation. ^*^P < 0.05, coated MCT vs. untreated MCT, Wilcoxon signed-rank test; ^#^*P* < 0.05, vs. LCT, Wilcoxon signed-rank test (Bonferroni correction). MCT, medium-chain triglycerides; LCT, long-chain triglycerides; AUC, area under the curve; ΔAUC, AUC of Δvalue; *C*_max_, maximum concentration; *ΔC*_max_, *C*_max_ of Δvalue; *T*_max_, time to reach *C*_max_.

### Secondary outcomes

#### Ketone bodies.

**Fig 2:**
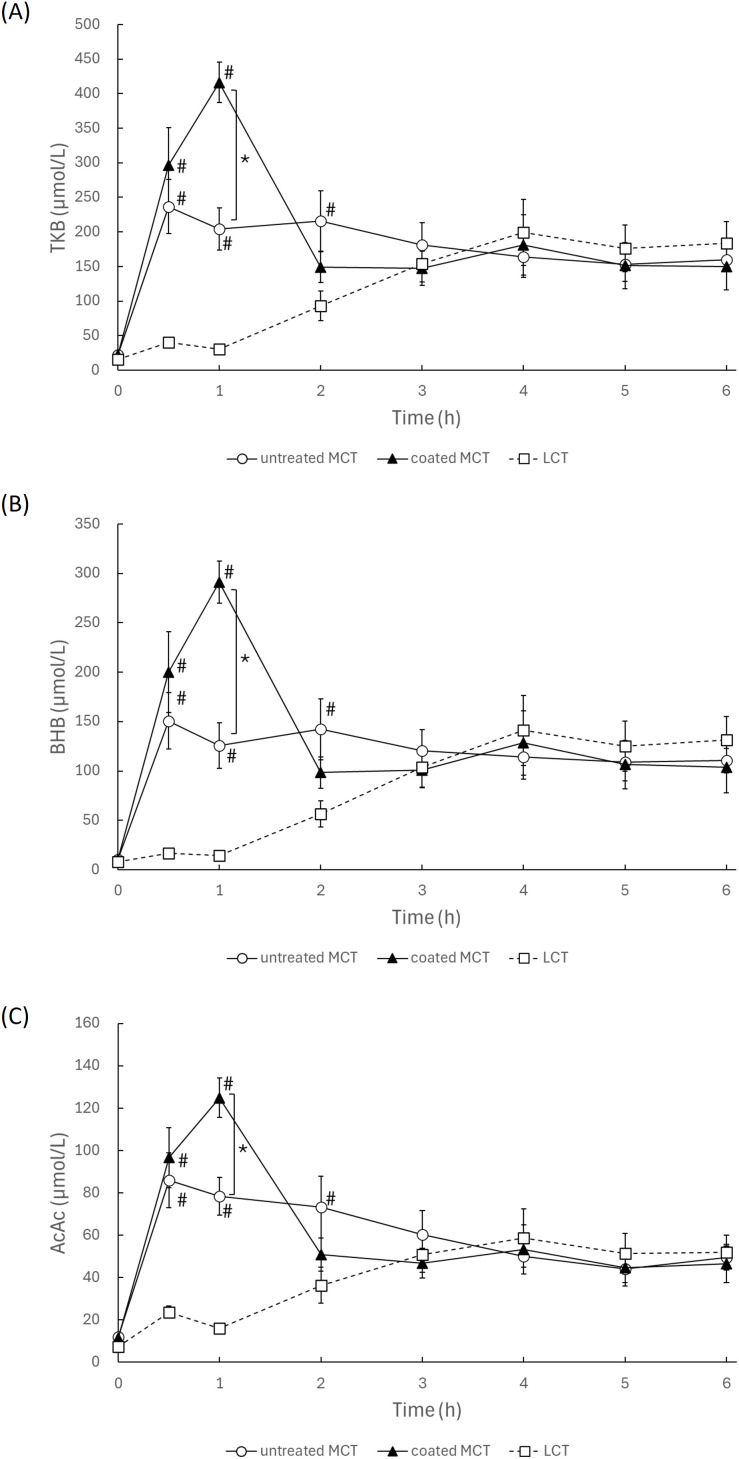
Time courses of ketone bodies. Time courses of (**A**) TKB, (**B**) BHB, and (**C**) AcAc are shown. Values are presented as means ± standard deviation. ^*^P < 0.05, coated MCT vs untreated MCT, Wilcoxon signed-rank test; ^#^*P* < 0.05, vs LCT, Wilcoxon signed-rank test (Bonferroni correction). TKB, total ketone bodies; BHB, β-hydroxybutyrate; AcAc, acetoacetate; MCT, medium-chain triglycerides; LCT, long-chain triglycerides.

As shown in Table 3 and Fig 2A, TKB increased from 0.5 h after ingesting both untreated MCT and coated MCT. At 1 h after ingestion, the level of coated MCT was significantly higher than that of untreated MCT (*P* = 0.003). *C*_max_ and Δ*C*_max_ were also significantly higher in coated MCT than in untreated MCT (both: *P* = 0.008). Meanwhile, *T*_max_ was significantly lower in coated MCT than in untreated MCT (*P* = 0.012). As for AUC, no significant difference was observed.

In LCT, TKB gradually increased after 2 h and then reached the same level as the two MCTs after 3 h. *C*_max_ and Δ*C*_max_ were significantly higher in coated MCT than in LCT (*P* = 0.036 and 0.045, respectively). *T*_max_ was significantly lower in coated MCT and untreated MCT than in LCT (*P* = 0.007 and 0.023, respectively). At 0.5 and 1 h after ingestion, coated MCT was significantly higher than LCT (*P* < 0.05). Untreated MCT was also significantly higher than LCT at 0.5–2 h after ingestion (*P* < 0.05).

BHB and AcAc showed similar results to TKB (Tables 4, 5, and Figs 2B and C). In both MCTs, BHB and AcAc increased from 0.5 h after ingestion. At 1 h after ingestion, coated MCT showed significantly higher values than untreated MCT (*P* < 0.05). The *C*_max_ and Δ*C*_max_ of BHB were also significantly higher in coated MCT than in untreated MCT (both: *P* = 0.003). Additionally, *T*_max_ was significantly lower in coated MCT than in untreated MCT (*P* = 0.012). AUC and ΔAUC showed no significant differences between the groups.

**Table 4:** Pharmacokinetic parameters of β-hydroxybutyrate.

	Unit	untreated MCT	coated MCT	LCT
**AUC**_**0-6h**_	μmol/L·h	713 ± 280	807 ± 328	513 ± 320
**ΔAUC**_**0-6h**_	μmol/L·h	652 ± 282	743 ± 341	465 ± 311
***C***_**max**_	μmol/L	225 ± 89	316 ± 97*^#^	173 ± 111
**Δ*C***_**max**_	μmol/L	215 ± 89	306 ± 99*	165 ± 110
***T***_**max**_	h	2.4 ± 1.6^#^	1.2 ± 1.0*^#^	4.3 ± 1.0

Values are presented as means ± standard deviation. *P < 0.05, coated MCT vs. untreated MCT, Wilcoxon signed-rank test; ^#^*P* < 0.05, vs. LCT, Wilcoxon signed-rank test (Bonferroni correction). MCT, medium-chain triglycerides; LCT, long-chain triglycerides; AUC, area under the curve; ΔAUC, AUC of Δvalue; *C*_max_, maximum concentration; *ΔC*_max_, *C*_max_ of Δvalue; *T*_max_, time to reach *C*_max_.

**Table 5:** Pharmacokinetic parameters of acetoacetate.

	Unit	untreated MCT	coated MCT	LCT
**AUC**_**0-6h**_	μmol/L·h	357 ± 123	364 ± 138	249 ± 140
**ΔAUC**_**0-6h**_	μmol/L·h	285 ± 117	295 ± 135	204 ± 131
***C***_**max**_	μmol/L	106 ± 40^#^	130 ± 36^#^	71 ± 44
**Δ*C***_**max**_	μmol/L	94 ± 37^#^	119 ± 34^#^*	63 ± 42
***T***_**max**_	h	1.9 ± 1.8^#^	1.1 ± 1.0^#^	4.2 ± 1.3

Values are presented as means ± standard deviation. ^*^P < 0.05, coated MCT vs. untreated MCT, Wilcoxon signed-rank test; ^#^*P* < 0.05, vs. LCT, Wilcoxon signed-rank test (Bonferroni correction). MCT, medium-chain triglycerides; LCT, long-chain triglycerides; AUC, area under the curve; ΔAUC, AUC of Δvalue; *C*_max_, maximum concentration; *ΔC*_max_, *C*_max_ of Δvalue; *T*_max_, time to reach *C*_max_.

### MCFAs

**Fig 3:**
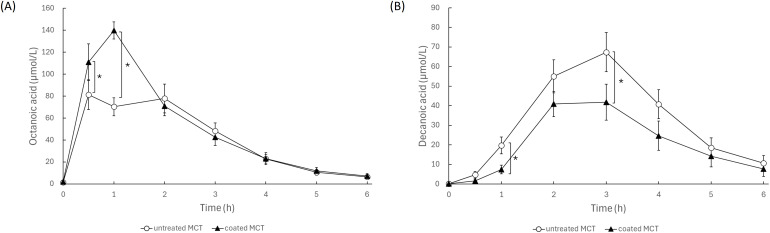
Time courses of MCFAs. Time courses of (**A**) octanoic acid and (**B**) decanoic acid are shown. Values are presented as means ± standard deviation. ^*^P < 0.05, coated MCT vs. untreated MCT, Wilcoxon signed-rank test. The octanoic acid and decanoic acid levels after LCT ingestion were all below the detection limit. MCFAs, medium-chain fatty acids; MCT, medium-chain triglycerides.

Octanoic acid increased from 0.5 h after ingestion in untreated and coated MCTs, as did ketone bodies (Fig 3A). At 0.5 and 1 h after ingestion, octanoic acid concentration was significantly higher in coated MCT than in untreated MCT (*P* = 0.026 and 0.003, respectively). AUC and ΔAUC were also significantly higher in coated MCT than in untreated MCT (*P =* 0.033 and 0.021, respectively; Table 6). Furthermore, coated MCT had significantly higher *C*_max_ and Δ*C*_max_ than untreated MCT (*P* = 0.010 and 0.008, respectively). Meanwhile, *T*_max_ was significantly lower in coated MCT than in untreated MCT (*P* = 0.036).

**Table 6:** Pharmacokinetic parameters of octanoic acid.

	Units	untreated MCT	coated MCT
**AUC**_**0-6h**_	μmol/L·h	256.7 ± 67.8	313.1 ± 73.6^*^
**ΔAUC**_**0-6h**_	μmol/L·h	247.6 ± 63.9	300.5 ± 74.7^*^
***C***_**max**_	μmol/L	111.3 ± 34.7	154.2 ± 37.5^*^
**Δ*C***_**max**_	μmol/L	109.8 ± 34.6	152.1 ± 38.7^*^
***T***_**max**_	h	1.68 ± 0.93	1.00 ± 0.39^*^

Values are presented as means ± standard deviation. ^*^P < 0.05, coated MCT vs. untreated MCT. Wilcoxon signed-rank test. Octanoic acid levels after LCT ingestion were all below the detection limit. MCT, medium-chain triglycerides; LCT, long-chain triglycerides; AUC, area under the curve; ΔAUC, AUC of Δvalue; *C*_max_, maximum concentration; *ΔC*_max_, *C*_max_ of Δvalue; *T*_max_, time to reach *C*_max_.

Unlike ketone bodies and octanoic acid, decanoic acid gradually increased until 3 h in both MCTs (Fig 3B). In addition, coated MCT showed lower levels than untreated MCT. At 1 and 3 h after ingestion, the level of coated MCT was significantly lower than that of untreated MCT (*P* = 0.041 and 0.033, respectively). The coated MCT also had significantly lower *C*_max_ and Δ*C*_max_ than untreated MCT (both: *P* = 0.021; Table 7). Moreover, AUC, ΔAUC, and *T*_max_ showed no significant differences (*P* > 0.05). In LCT, blood octanoic acid and decanoic acid were all below the detection limit.

**Table 7:** Pharmacokinetic parameters of decanoic acid.

	Units	untreated MCT	coated MCT
**AUC**_**0-6h**_	μmol/L·h	204.1 ± 106.1	131.8 ± 98.5
**ΔAUC**_**0-6h**_	μmol/L·h	203.5 ± 105.0	130.6 ± 97.2
***C***_**max**_	μmol/L	70.4 ± 32.0	46.5 ± 27.8^*^
**Δ*C***_**max**_	μmol/L	70.3 ± 31.7	46.3 ± 27.6^*^
***T***_**max**_	h	2.8 ± 0.4	2.5 ± 0.5

Values are presented as means ± standard deviation. ^*^P < 0.05, untreated MCT vs. coated MCT, Wilcoxon signed-rank test. Decanoic acid levels after LCT ingestion were all below the detection limit. MCT, medium-chain triglycerides; LCT, long-chain triglycerides; AUC, area under the curve; ΔAUC, AUC of Δvalue; *C*_max_, maximum concentration; *ΔC*_max_, *C*_max_ of Δvalue; *T*_max_, time to reach *C*_max_.

### Correlation between TKB and MCFAs

**Fig 4:**
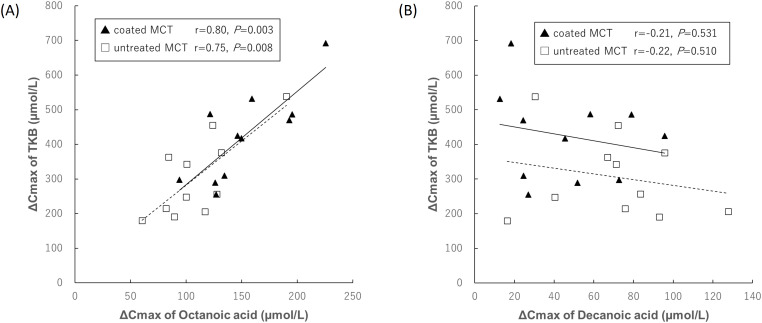
Relationship between TKB and MCFAs. MCFAs, medium-chain fatty acids; TKB, total ketone bodies.

Fig 4 shows the relationships between the Δ*C*_max_ of TKB and the Δ*C*_max_ of MCFAs. Octanoic acid showed a relatively strong and significantly positive correlation with TKB in both coated and untreated MCT (*r* = 0.80, *P* = 0.003; *r* = 0.75, *P* = 0.008). Conversely, decanoic acid showed no correlation with TKB.

### Ghrelin

Active ghrelin and desacyl ghrelin did not significantly change after ingestion (S4 Fig). At 6 h after ingestion, desacyl ghrelin levels were significantly lower in coated MCT than in untreated MCT (*P* = 0.033) and were significantly higher in untreated MCT than in LCT (*P* = 0.049).

### Gastrointestinal symptoms

The frequency of symptoms such as stomachache, indigestion, nausea, bloating, and fullness were low, with no significant differences observed between the groups (S5 Fig).

### Safety

Table 8 lists the frequency of adverse events. All were mild symptoms. The presence of adverse events was not significantly different between the groups (*P* = 1.000).

**Table 8:** Adverse events.

	Untreated MCT	Coated MCT
**Adverse events**	10 events (4 subjects)	14 events (5 subjects)
**Specific symptoms**	indigestion (2 events)	indigestion (4 events)
	stomachache (2 events)	stomachache (3 events)
	nausea (1 event)	nausea (1 event)
	bloating (2 events)	bloating (3 events)
	fullness (3 events)	fullness (3 events)

MCT, medium-chain triglycerides; LCT, long-chain triglycerides.

## Discussion

This study was conducted with the hypothesis that coating the MCT with protein would allow MCT to reach the small intestine in microparticles and enhance the ketogenic effect. The primary outcome, i.e., the ΔAUC of TKB, did not significantly differ between MCTs with and without coating. However, as the secondary outcome, the Δ*C*_max_ of TKB, BHB, AcAc, and octanoic acid were significantly higher in coated MCT than in untreated MCT.

In this study, ΔAUC, an index of total exposure to ketone bodies over a defined period, was selected as the primary endpoint to evaluate the ketogenic effect. One possible explanation for the lack of observed efficacy with ΔAUC is the influence of endogenous ketogenesis. In this study, ΔAUC was calculated using plasma concentrations measured up to 6 h after intake. However, after 3 h, the prolonged fasting period may have induced endogenous ketogenesis, potentially diluting the ketogenic effect of MCT intake. Indeed, after 3 h, TKB levels increased above baseline in all groups, including the LCT group, and the differences between groups disappeared (Fig 2). To more accurately evaluate the acute ketogenic effect of MCTs, the ΔAUC calculated over a shorter time window may be a better reflection.

The Δ*C*_*max*_ results suggest that coating the MCT may enhance ketogenesis by increasing octanoic acid absorption. The mechanism of this effect may be that the coated MCT is resistant to gastric acid, allowing it to reach the small intestine in the form of microparticles and to be highly absorbable. β-lactoglobulin, one of the milk proteins, can be used to coat some bioactive compound to improve its bioavailability [26]. This protein is resistant to digestion by pepsin in the stomach, but it is digested by trypsin in the small intestine, owing to its structural characteristics [27]. Given that the coating material used in this study (milk protein) also contains β-lactoglobulin, its differential sensitivity to digestive enzymes may contribute to the increased octanoic acid absorption and ketogenesis.

The increase in Δ*C*_*max*_, namely the peak TKB concentration observed following the coating treatment, is particularly significant in the context of dietary interventions targeting cognitive function. Previous studies have reported that, in patients with Alzheimer’s disease, cerebral glucose utilization is reduced, whereas the capacity to utilize ketone bodies remains relatively intact [28]. Under such pathological conditions, the efficacy of MCTs has been suggested. Indeed, prior studies in patients with mild cognitive impairment or Alzheimer’s disease have shown that increases in peak plasma ketone body concentrations following a single MCT intake are positively correlated with improvements in cognitive performance [17, 29, 30]. These findings therefore suggest that coating MCTs may further enhance cognitive function by boosting the peak ketone response.

In this study, TKB had a relatively strong correlation with octanoic acid, consistent with previous studies [22, 24, 31]. The conversion efficiency of octanoic acid to ketone bodies is higher than that of decanoic acid [3, 32]. As in previous studies [22, 24, 31], we used MCT containing more octanoic acid than decanoic acid. These factors may have led to the strong correlation between ketone body synthesis and octanoic acid absorption.

The peak of decanoic acid elevation was later than that of octanoic acid, consistent with similar previous studies [22, 31]. Given that triglyceride hydrolytic activity by lipase is higher with shorter chain length [33], decanoic acid may be hydrolyzed more slowly than octanoic acid. In addition, both octanoic acid and decanoic acid are partially absorbed via the portal vein, although a higher proportion of decanoic acid is absorbed via the lymphatic vessels [34–36]. These differences in hydrolysis rates and absorption pathways might explain the differences in the results of decanoic acid and octanoic acid. Moreover, the effect of coating on blood decanoic acid was opposite to that of blood octanoic acid. Although its mechanism is unknown, the differences in hydrolysis rate and absorption pathway may also affect this result. Further research is needed for confirmation.

In this study, the content of protein, fat, and carbohydrate in each test food was the same. However, leucine derived from milk protein in the test foods may have raised the ketogenic effect in both groups. Under certain conditions, leucine causes ketogenesis in rat liver cells [37]. In rat-derived astroglial cells, leucine is metabolized to ketone bodies and used as energy in neurons and other cells [38]. Thus, coating with milk proteins containing leucine may also promote ketogenesis.

Long-term MCT intake activates ghrelin [12–14], possibly contributing to the effects of MCT on cognitive function and muscle strength along with ketogenesis. To date, the effect of a single MCT intake on ghrelin is still insufficiently studied. In a previous study [2], however, total ghrelin tended to decrease up to 3 h after a single intake, reflecting the fact that ghrelin decreases rapidly after meal intake [39]. In the present study, the levels of active ghrelin and desacyl ghrelin did not significantly change in any group up to 6 h after ingestion. The decrease in ghrelin immediately after meal intake may not have occurred because of the low energy intake from the test foods. The effect of long-term consumption of coated MCT on ghrelin activation requires further investigation.

MCT intake is known to cause gastrointestinal symptoms [15, 22-24], and a certain number of adverse events occurred in this trial. No significant differences in incidence were noted between the groups, and all events were mild, suggesting that coated MCTs are safe to consume. However, the efficacy of coated MCT on gastrointestinal symptoms, another hypothesis, could not be confirmed. In one report on the gastrointestinal symptoms of MCT [22], 10–30 g of MCTs were consumed. In the present study, the primary outcome was the ketogenic effect, and the dose of MCTs was set at 6 g, which is in line with the doses used in cognitive function and physical function studies [19]. Studies with higher doses of coated MCT are necessary to evaluate the efficacy on gastrointestinal symptoms.

This study has several limitations. First, this study was conducted with a small sample size because it was a pilot study. The small sample size may have limited the statistical power to detect between-group differences in ΔAUC. Additionally, the selected time window for ΔAUC calculation may not have been optimal to fully capture the acute ketogenic response. Collectively, these methodological factors may have contributed to the absence of statistically significant differences in ΔAUC. Therefore, validation with a larger sample size and reconsideration of the ΔAUC calculation method are warranted in future studies. Second, this study included only healthy young to middle-aged men. It is necessary to be careful not to extrapolate the results directly to women or other age groups, as sex differences and aging may affect lipid metabolism and ketone body kinetics. Future studies with a more diverse range of subjects are also required. Finally, this study evaluated the acute effects of a single ingestion. The effects and safety of long-term ingestion are the subject of future studies. Despite these limitations, this study suggests for the first time that protein coating can enhance the ketogenic effect of MCT.

## Conclusions

The ΔAUC in TKB, which was the primary outcome, showed no significant differences between MCTs with and without coating treatment. However, coated MCTs showed significant effects on the ΔCmax of TKB, BHB, AcAc, and octanoic acid as the secondary outcome. Furthermore, TKB exhibited a significant positive correlation with octanoic acid. Therefore, MCT coating with milk protein may increase the ketogenic effect by enhancing the absorption of MCFAs, especially octanoic acid.

## Supporting information

S1 File CONSORT 2010 checklist. (PDF)

S2 File Translated study protocol. (PDF)

S3 Fig Microscopic images of coated MCT. (DOCX)

S4 Fig Time courses of active ghrelin and desacyl ghrelin. (DOCX)

S5 Fig Time courses of gastrointestinal symptoms. (DOCX)

S6 Table Details of participants’ background. (DOCX)

S7 File Data of study. (XLSX)
