# Supplementary material for: Ketogenic effects of milk protein–coated medium-chain triglycerides in healthy men: A randomized, double-blind, crossover study
Source: PLoS One. 2026 Aug 10;21(8):e0355475. doi: 10.1371/journal.pone.0355475 (PMC13456251; doi:10.1371/journal.pone.0355475)
Supplement: Supplementary file 2 [file pone.0355475.s002.pdf]

Research protocol

The effect of milk protein coating on the absorption of medium-chain triglycerides.

Protocol No: FA6-2309

(Research identification number: 2023 – 006)

Date of preparation: December 15, 2023

Responsible organization: Meiji Co., Ltd.

Edition number: Edition 1

List of abbreviations

Abbreviations	Name not abbreviated
AUC	Area Under the Curve
ΔAUC	Delta Area Under the Curve
Cmax	Maximum Concentration
ΔCmax	Delta Maximum Concentration
Tmax	Maximum Concentration Time
MCT	Medium-Chain Triglycerides
LCT	Long-chain Triglycerides
BMI	Body Mass Index
SCR	Screening
UMIN-CTR	University Hospital Medical Information Network-Clinical Trials Registry
AST	Aspartate Aminotransferase
ALT	Alanine Aminotransferase
γ-GTP	γ-Glutamyl Transpeptidase
ALP	Alkaline Phosphatase
LDH	Lactate Dehydrogenase
HDL	High-Density Lipoprotein
LDL	Low-Density Lipoprotein
HbA1c	Hemoglobin A1c

1. **Outline of the research plan**

Research name	The effect of milk protein coating on the absorption of medium-chain triglycerides.<br>Protocol No: FA6-2309 (ID: 2023 - 006)
Objective	The purpose of this study was to investigate the absorption of a single ingestion of milk protein-coated MCT-containing food in comparison with untreated MCT-containing food in healthy adults.
Study design	Randomized, double-blind, crossover comparison
Objective Number of subjects	12 (6 subjects in 1 group ×2 groups)
Study period	December 2023 to March 2024<br>(Ethics review committee approval to data set fixation)
Study subjects	< Inclusion criteria ><br>1) Subjects who were fully informed of the purpose and content of the study, were able to give consent, and fully understood the information before freely volunteering and consenting to participate<br>2) Men aged 20 to less than 40 years at the time of obtaining consent<br>3) Subjects with a BMI of 18.5 to less than 25.0 kg/m^2<br>Exclusion criteria<br>1) Subjects who took drugs, quasi-drugs, supplements, foods with health function (Food with nutritional function, food for specified health use, food with functional claims), or MCT or coconut oil (including foods containing many of them) for at least 2 days a week for 1 month before the screening test, or who planned to take them during the study period, in the opinion of the principal investigator as having a potential impact on the study<br>2) Subjects who were judged by the principal investigator as having a serious chronic disease or symptom<br>3) Subjects who were judged by the principal investigator as having abnormal lipid metabolism, glucose metabolism, liver function, or kidney function<br>4) Those with food allergy<br>5) Those with lactose intolerance<br>6) Those who have undergone gastrointestinal surgery in the past<br>7) Those who smoked in the 1 month prior to the screening test<br>8) Those who donated 200 mL of blood in the 1 month prior to the screening

	test or 400 mL in the 3 months prior to the screening test<br>9) Those who participated in other clinical or monitoring studies in the 1 month prior to the screening test, or those who plan to participate in other clinical studies during the study period<br>10) Other subjects judged by the investigator to be inappropriate subjects
Food under study	Control food: Powdered beverage containing MCT without milk protein coating (contains 6 g of MCT)<br>Test food: Powdered beverage containing MCT coated with milk protein (contains 6 g of MCT)<br>Reference food: powdered beverage in which LCT was substituted for MCT in the control food
Intake method	Each study food should be consumed once on each test day. A washout period of at least 6 days should be allowed between test days. Study food should be consumed within 2 minutes if possible.
Endpoints	• Primary endpoint<br>• Blood total ketone body (ΔAUC)<br>• Secondary endpoint<br>• Blood metabolites and metabolic markers (AUC, ΔAUC, Cmax, ΔCmax, Tmax)<br>✧ Blood ketone bodies (β-Hydroxybutyric acid, acetoacetic acid, and the sum)<br>✧ Medium-chain fatty acids (Octanoic acid and decanoic acid)<br>✧ Acylated ghrelin and desacylghrelin<br>✧ Glucose<br>✧ Insulin<br>• Abdominal symptom questionnaire<br>✧ Graded scale for stomachache, heavy feeling, nausea, bloating, and appetite<br>• Safety (incidence of adverse events and adverse drug reactions)

2. Contents

1. Outline of the research plan 3

2. Contents 5

3. Background Information 8

4. Objective of this study 8

5. Endpoints 8

5.1. Primary endpoint 8

5.2. Secondary endpoints 8

5.3. Safety endpoints 9

6. Study design 9

6.1. Target number of subjects 9

6.2. Rationale for setting the number of subjects 9

6.3. Study period 9

7. Subjects 9

7.1. Inclusion criteria 9

7.2. Exclusion criteria 10

8. Food subject to research 10

8.1. Contents of food subject to research 10

8.1.1. Test food 10

8.1.2. Control food 10

8.1.3. Reference food 11

8.2. Intake method and duration 11

8.3. Rationale for setting the intake 11

8.4. Confirmation of blinding 11

8.5. Management of the food under study 11

9. Schedule, observation, inspection, and survey items and procedures 12

9.1. Schedule, observation, inspection, and survey items 12

9.1.1. Research schedule 12

9.1.2. Details of observation, examination, and survey items 14

9.1.3. Implementation method for each observation, examination, and survey item 15

9.2. Flow of Research 16

9.2.1. Recruitment of Study Subjects 16

9.2.2. Anthropometry 16

9.2.3. Pre-stay examination 16

9.2.4. Obtaining Consent 16

9.2.5. Screening Test 1 (SCR1) 16
9.2.6. Selection of research subjects 16
9.2.7. Selection of research subjects 1 16
9.2.8. Screening Test 2 (SCR2) 17
9.2.9. Selection of Study Subjects 2 17
9.2.10. Allocation 17
9.2.11. Phase I Study 17
9.2.12. Phase 2 Research 17
9.2.13. Restrictions on Research Subjects during the Study Period 18
9.2.13.1. Prohibited items 18
9.2.13.2. Precautions 18
10. Preparation of case report form or data list 19
11. Determination of subjects for analysis 19
12. Key open 19
13. Statistical analysis 19
13.1. Analysis set 19
13.2. Evaluation and Analysis 20
13.2.1. Evaluation and analysis of primary and secondary endpoints 20
13.2.2. Safety assessment and analysis 20
14. Adverse events 20
14.1. Definition of adverse events 20
14.2. Definition of a serious adverse event 21
14.3. Collection and recording of adverse events 21
15. Ensuring the safety of research subjects 22
15.1. Basic matters 22
15.2. Response in the event of a serious adverse event 23
16. Burden and expected risks and benefits for research subjects 23
16.1. Burden and expected risks for research subjects 23
16.1.1. Events caused by ingestion of the study food 23
16.1.2. Physical distress and risk of infection associated with blood sampling 23
16.1.3. Mental distress associated with measurements and investigations 24
16.2. Projected benefits 24
16.3. Comprehensive Assessment and Measures to Minimize Burden and Risk 24
17. Withdrawal and dropout of individual subjects 24
17.1. Definition 24
17.2. Discontinuation criteria for individual research subjects 25

17.3. Discontinuation procedure for individual research subjects ... 25
17.4. Response to dropout ... 25
18. Discontinuation of the entire research ... 25
19. Adherence to, revision of, deviation from, or modification of the research protocol ... 26
19.1. Agreement and adherence to the research protocol ... 26
19.2. Revision of the research protocol ... 26
19.3. Deviation from the research protocol ... 27
20. Ethical conduct of research ... 27
20.1. Ethical review and permission or report from the head of the research institution 27
20.1.1. Compliance with ethical guidelines ... 27
20.1.2. Review by the ethical review committee and permission from the director of the research institution ... 27
20.1.3. Report to the head of the research institution ... 27
20.1.4. Report to the ethics review committee ... 28
20.2. Consent for research participation ... 28
20.2.1. Contents to be included in the explanatory consent document ... 28
20.2.2. Timing and Method of Obtaining Consent ... 28
20.2.3. Points of Attention Regarding Obtaining Consent ... 29
20.2.4. Revising the Consent Explanatory Document and the Consent Form ... 29
20.3. Matters Related to the Protection of Human Rights of Research Subjects ... 29
20.3.1. Basic Matters ... 29
20.3.2. Management of Identification and Correspondence Table ... 29
20.4. Compensation and Insurance for Health Damage ... 30
20.5. Matters related to incidental findings obtained in this research ... 30
21. Storage and disposal of samples and information obtained in this research ... 30
21.1. Storage and Disposal of Samples Obtained in the Study ... 30
21.2. Storage and disposal of information and records ... 30
21.3. Creation of records concerning the transfer of samples and information ... 31
23. Direct access of source documents, etc. ... 31
24. Monitoring ... 31
25. Conflicts of interest ... 31
26. Registration of the outline, progress, and results of this study ... 32
27. References ... 32

3. Background Information

Medium-chain fats and oils (MCT) are fats and oils composed of medium-chain fatty acids (typically fatty acids with 8 to 12 carbon atoms). Medium-chain fatty acids are also found in foods such as coconut oil and milk. It is known that MCT is more rapidly absorbed and converted to ketone bodies than common long-chain fatty acids and fats (LCT) [2]. Ketone bodies are a generic term for acetone, acetoacetate, and β-hydroxybutyrate. Like glucose, ketone bodies are used as energy in brain cells [3] and skeletal muscle. Because of these characteristics, many functional studies have been conducted on MCT, and its usefulness in improving cognitive function [4,5] in the elderly, muscle strength in the elderly [6], and muscle mass in athletes [7] has been reported. Ghrelin, an appetite-promoting and anabolic hormone, is activated by binding to octanoic acid, a medium-chain fatty acid. It has been reported that intake of MCT increases activated ghrelin [8-10], which may partly contribute to the above functions of MCT.

Abdominal symptoms such as stomachache, upset stomach, and diarrhea are known to occur when a large amount of MCT is consumed [1]. The hypothesis for the mechanism of abdominal symptoms of MCT is that MCT, unlike LCT, is easily degraded by gastric lipase, and the released medium-chain fatty acids stimulate gastric tissue. Therefore, we developed MCT coated with milk protein so that MCT is not easily degraded by gastric lipase. This coating treatment can easily reduce abdominal symptoms. On the other hand, there is a concern that the coating treatment with milk protein may affect absorption in the gastrointestinal tract, but this has not been investigated.

4. Objective of this study

The purpose of this study was to investigate the absorption of milk protein-coated MCT foods compared with uncoated MCT foods after a single ingestion in healthy adults.

5. Endpoints

5.1. Primary endpoint

・Blood total ketone body (ΔAUC)

5.2. Secondary endpoints

- Blood metabolites and metabolic markers AUC, ΔAUC, Cmax, ΔCmax, and Tmax
  - ➢ Blood ketone body (β-Hydroxybutyric acid, acetoacetic acid, and the sum)
  - ➢ Medium-chain fatty acids (Octanoic acid and decanoic acid)
  - ➢ Acylated ghrelin and desacylghrelin
  - ➢ Glucose
  - ➢ Insulin
- Abdominal Symptoms Questionnaire
  - ➢ Stomach pain, heavy feeling, nausea, bloating, appetite graded

5.3. Safety endpoints

・Number, number, and frequency of adverse events

・Number, number, and frequency of study-related adverse events

6. Study design

Randomized, double-blind, crossover comparison

6.1. Target number of subjects

12 subjects (6 subjects in 1 group ×2 groups)

6.2. Rationale for setting the number of subjects

Since there is no previous study on the evaluation of the absorbability of MCT coated with milk protein, we set the number of subjects to 12 by referring to the similar literature below.

- In a crossover study comparing increases in blood ketone bodies and medium-chain fatty acids and the development of abdominal symptoms with the ingestion of unemulsified or emulsified MCT, it was confirmed that emulsification significantly increased blood ketone bodies and reduced abdominal symptoms in 10 subjects [1].
- In a crossover study comparing blood ketone bodies, total ghrelin, and gastric emptying with the ingestion of MCT, vegetable oils, and conjugated linolenic acid, it was confirmed that MCT significantly increased blood ketone bodies and delayed gastric emptying compared with vegetable oils in 15 subjects [11].

6.3. Study period

December 2023 to March 2024 (from ethical review committee approval to data set fixation)

7. Subjects

7.1. Inclusion criteria

1) Applicants who have been fully informed of the purpose and content of the study, have the capacity to consent, and fully understand the purpose and content of the study, and have voluntarily volunteered and consented to participate.
2) Men aged 20 to less than 40 years at the time of obtaining consent
3) Subjects with a BMI of 18.5 to less than 25.0 kg/m^2

7.2. Exclusion criteria

1) Subjects who took drugs, quasi-drugs, supplements, health functional foods (Food with nutritional function, food for specified health use, food with functional claims), or MCT or coconut oil (including foods containing many of them) at least 2 days a week for 1 month before the screening test, or who planned to take them during the study period.
2) Subjects who were judged by the investigator to have serious chronic diseases or symptoms.
3) Subjects who were judged by the investigator to have abnormal lipid metabolism, glucose metabolism, liver function, or kidney function.
4) Subjects who have food allergies.
5) Subjects who have lactose intolerance.
6) Subjects who had undergone digestive system surgery in the past.
7) Subjects who smoked in the month before the screening test.
8) Subjects who donated 200 mL of blood within 1 month or 400 mL within 3 months prior to the screening test
9) Subjects who participated in other clinical or monitoring studies within 1 month prior to the screening test, or who plan to participate in other clinical studies during the study period
10) Other subjects judged by the investigator to be inappropriate as study subjects

[Basis for setting exclusion criteria]

1), 2), 3), 6), 7), 9) Due to influence on efficacy evaluation

4), 5), 8), 10) Due to consideration for safety of study subjects

8. Food subject to research

8.1. Contents of food subject to research

8.1.1. Test food

Food containing MCT coated with milk protein

Shape and formPowdered beverage

Intake : 18.9 g/sachet (6 g/sachet as MCT)

Method of use : Take 1 sachet at a time on each evaluation day.

Quality retention period: 90 days

8.1.2. Control food

Food containing MCT without milk protein coating

Shape and formPowdered beverage

Intake : 18.9 g/sachet (6 g/sachet as MCT)

Method of use : Take 1 sachet at a time on each evaluation day.

Quality retention period: 90 days

8.1.3. Reference food

Food in which LCT replaces MCT of control food

Shape and formPowdered beverage

Intake 18.9 g/sachet (LCT replaces MCT of control food)

Method of use Intake 1 sachet at 1 time on the day of SCR2.

Retention period: 90 days

*The reference food will be used for SCR2 selection.

8.2. Intake method and duration

Intake 1 sachet per test on each test day. At intake, use a shaker to mix the entire amount of 1 sachet of the study food with 200 m L of water and drink the entire amount. Add 50 mL of separately prepared water to the shaker, pour as if washing off the remaining liquid, and drink up. All these steps should be performed within 2 minutes.

8.3. Rationale for setting the intake

The efficacy of MCT 6 g on cognitive function [5] and muscle strength [6] in frail elderly people has been reported.

8.4. Confirmation of blinding

The allocation manager conducts an indistinguishability test of the food under study and confirms that the test food and the control food are indistinguishable.

8.5. Management of the food under study

The food under study manufacturing manager prepares an identification table of the food under study symbol and the food under study type (Test and control foods). The food under study manufacturing manager seals the identification table and sends it to the allocation manager through the research management organization. The allocation manager keeps the sealed identification table until key opening. The food subject to research should be stored away from direct sunlight and high temperature and humidity.

The research representative should send the food subject to research to the food subject manager. At this time, the research representative should make a record of the sending and the food subject manager should make a record of the receipt.

The food subject to research manager should make a food subject to research management table and manage the records of the distribution of the food subject to research, the number of stocks, and the

collection of the food subject to research.

The food subject to research remaining in the hands of the food subject to research manager after the research is completed should be disposed of by the food subject to research manager and the record should be entered in the food subject to research management table.

9. Schedule, observation, inspection, and survey items and procedures

9.1. Schedule, observation, inspection, and survey items

9.1.1. Research schedule

The SCR1, SCR2, Phase I, and Phase II studies will be conducted at least 6 days apart. On each examination day, overnight hospitalization will be conducted from the day before. The schedule of the entire study and the examination items on each examination day are shown in the chart below.

Schedule and examination items from the briefing session to SCR1

		Briefing session/SCR1 examination 2 days before	SCR1 examination day			
			Before ingestion	Time after ingestion (h)		Next day After
				0	1	
Obtained consent		○				
Anthropometry		○				
Pre-stay examination *1		○				
Background survey (SCR1)			○			
Vital signs			○			
Interview			○		○	
Selection of study subjects			○			
Abdominal symptom questionnaire			○		○	
Blood sampling *2	General blood tests		○			
	Blood metabolites and metabolic markers (blood ketone bodies only)		○		○	
Control food intake *3				○		
Confirmation of physical condition *4						○

*(a) Do not perform this test if the physical measurement does not meet the inclusion criteria

3).
*(b) Perform this test on persons who are judged by the physician to have no problem with blood sampling.
*(c) Drink up within 2 minutes if possible.
*(d) On or after the next day, confirm the physical condition and the presence of adverse events by telephone or e-mail.

Schedule and examination items for each study day (SCR2, Tier 1, and Tier 2 studies)

	Each examination 2 days before	Before ingestion	Time after ingestion (h)								on or after the following day and later
			0	0.5	1	2	3	4	5	6	
Pre-Accommodation Examination	○										
Background Survey (day of examination)		○									
Vital signs		○									
Interview		○								○	
Abdominal symptom questionnaire		○		○	○	○	○	○	○	○	
Blood sampling (blood metabolites/metabolic markers)		○[※1]		○	○	○[※1]	○	○[※1]	○	○[※1]	

Study food intake *2			○								
Check physical condition *3											○

*(a) Measurement of acylated ghrelin and desacylghrelin should be performed only before and 2, 4, and 6 hours after ingestion.

*(b) Drink up within 2 minutes. The reference food should be consumed in the SCR2 study, and the test food or control food should be consumed according to the allocation in the Phase I and Phase II studies.

*(c) Confirmation of physical condition and presence of adverse events should be made by telephone or e-mail on or after the next day.

9.1.2. Details of observation, examination, and survey items

Examination and survey	Items and details
Anthropometry	Height, weight, BMI
Pre-Accommodation Testing	PCR Testing (Confirmation of COVID-19 Infection)
Background Survey (SCR1)	Date of birth and age, sex, medical history (including presence or absence of digestive system surgery), current medical history, intake status of drugs, quasi-drugs, supplements, health functional foods, and MCT-containing foods, presence or absence of food allergy, presence or absence of lactose intolerance, smoking habit, blood donation status, presence or absence of overseas travel, presence or absence of participation in other studies, compliance with dietary and drinking restrictions on the day before the test, and confirmation of intake of a regulated diet and fasting
Background Survey (On the day of the test)	Compliance with restrictions on meals, drinks, and favorite foods, compliance with restrictions on drugs and health functional foods, changes in physical condition, changes in lifestyle habits, blood donation status, compliance with dietary and drinking restrictions on the day before the test, and confirmation of intake of a regulated diet and fasting
Interview	Subjective symptoms and objective findings
Vital signs	Systolic blood pressure, diastolic blood pressure, pulse, body temperature
General blood tests	Hematological tests<br>White blood cell count, red blood cell count, hemoglobin, hematocrit, platelet count

	Blood chemistry tests<br>Total protein, albumin, AST, ALT, γ-GTP, LDH, T-Bil, ALP, urea nitrogen, creatinine, uric acid, sodium, chloride, potassium, calcium, neutral fat, total cholesterol, HDL-cholesterol, LDL-cholesterol, HbA1c, HBs antigen, HCV antibody, HIV antigen/antibody, qualitative test for syphilis (TP antibody test)
Blood metabolites and metabolic markers	Blood ketone bodies (β-Hydroxybutyric acid and acetoacetic acid), medium-chain fatty acids (Octanoic acid and decanoic acid), acylated ghrelin and desacylghrelin, glucose, insulin
Abdominal symptom questionnaire	Graded assessment of abdominal symptoms (Stomach pain, heavy stomach, nausea, bloating, appetite, etc.)

9.1.3. Implementation method for each observation, examination, and survey item

1) Background survey

Use the "Background survey (SCR1)" or "Background survey (examination day)" to conduct a survey based on self-input by the research subjects.

2) Subjective symptoms and objective findings

The investigator or subinvestigator will interview the subjects.

3) Height and weight

Measure according to the method specified by the institution.

4) BMI

Calculate using height and weight.

5) Vital signs

Measure according to the method specified by the institution.

6) Blood sample

・Blood sample volume: 30 mL for SCR1, 24 mL each for SCR2, Phase 1, and Phase 2 (total blood sample volume: 102 mL)

General blood tests will be measured only during SCR1.

Only medium-chain fatty acids (Octanoic acid and decanoic acid), acylated ghrelin, and desacylghrelin will be measured at the responsible institution, and other items will be measured at a laboratory measurement institution.

7) Abdominal Symptom Questionnaire

The degree of stomachache, heavy stomach, nausea, abdominal fullness, and appetite will be investigated on a graded scale based on self-input by study subjects using an evaluation form.

9.2. Flow of Research

9.2.1. Recruitment of Study Subjects

Collaborating institutions send out recruitment guidelines for research subjects.

9.2.2. Anthropometry

Anthropometry will be performed at a medical institution on the day before the SCR1 visit, and it will be confirmed whether the patient meets inclusion criteria 3).

Check whether or not it is.

9.2.3. Pre-stay examination

Test will be performed at a medical institution on the day before each visit. If the test result on the following day is positive, the patient will not be allowed to participate.

9.2.4. Obtaining Consent

Consent will be obtained in accordance with the [20.2. Consent to participate in research].

9.2.5. Screening Test 1 (SCR1)

Conduct according to the schedule, items, and methods described in [9.1. Schedule, items for observation, inspection, and investigation]. Enter the accommodation facility by the designated time on the previous day. The evening meal on the previous day shall be the designated meal and consumed by 21:00. After 21:00 on the previous day, participants shall be fasting except for the designated meal until the end of the examination, and only water shall be allowed. For breakfast on the day, participants shall eat the designated meal approximately 1.5~2.5 hours before the scheduled meeting time. The subject food (control food in SCR 1) shall be consumed by mid-morning.

The designated meal is intended to satisfy the dietary conditions of the research subjects and shall be as follows.

- Dinner on the previous day shall be curry rice and vegetable soup.
- Breakfast on the day shall be jelly drink containing sugar.
- The same menu shall be used in SCR2, Phase I, and Phase II.

9.2.6. Selection of research subjects

Based on the results of SCR1, the investigator shall select those who meet the criteria of [7.1. Selection Criteria] and do not fall under [7.2. Exclusion criteria].

9.2.7. Selection of research subjects 1

From the selected research subjects, the investigator shall exclude and select 18 subjects in the following manner.

- Based on the results of general blood tests in SCR1, those judged to be inappropriate as research subjects shall be excluded.
- In SCR1, 18 subjects shall be selected from those whose blood total ketone body increase before and 1 hour after intake of the control food was close to the median value as an index.

9.2.8. Screening Test 2 (SCR2)

This test shall be conducted at least 6 days after SCR1. This test shall be conducted according to the schedule, items, and methods described in [9.1. Schedule, items for observation, inspection, and investigation]. Enter the accommodation facility by the designated time on the previous day. The prescribed dinner on the previous day shall be consumed by 21:00. After 21:00 on the previous day, participants should not eat anything other than the designated meal until the end of the examination, and should only be allowed to drink water. On the day of the examination, participants should eat the designated breakfast approximately 1.5~2.5 hours before the scheduled meeting time. The subject food (reference food in SCR 2) should be consumed by mid-morning.

9.2.9. Selection of Study Subjects 2

From the subjects selected in SCR1, the principal investigator will select 12 subjects in the following manner.

- The ΔAUC of blood total ketone bodies before and 6 hours after the intake of the reference food in SCR2 will be calculated, and 12 subjects will be selected from those with the smallest change.

9.2.10. Allocation

The allocation manager will allocate the selected subjects to 2 groups based on age and the increase in blood total ketone bodies in SCR1 using the stratified permuted block method so that there are no differences between groups, and prepare an allocation table.

After completing the allocation of subjects, the allocation manager will hand over the allocation table to the food subject manager through the research management organization. The food subject manager will strictly manage the allocation table. In the event of an emergency, such as the occurrence of a serious adverse event, only the minimum information will be disclosed.

9.2.11. Phase I Study

This study will be conducted at least 6 days after SCR2. This study will be conducted according to the schedule, items, and methods described in [9.1. Schedule, items for observation, inspection, and investigation]. Enter the accommodation facility by the designated time on the previous day. The dinner on the previous day will be the prescribed meal and consumed by 21:00. After 21:00 on the previous day, participants will be allowed to eat nothing other than the designated meal and drink only water until the end of the study. The breakfast on the day will be the prescribed meal and consumed approximately 1.5~2.5 hours before the scheduled meeting time. The food subject to the study will be consumed by mid-morning.

9.2.12. Phase 2 Research

This study will be conducted at least 6 days after the Phase I study. This study will be conducted according to the schedule, items, and methods described in [9.1. Schedule, items for observation, inspection, and investigation]. Enter the accommodation facility by the designated time on the previous day. The dinner on the previous day will be the prescribed meal and consumed by 21:00. After 21:00 on

the previous day, participants will be allowed to eat nothing other than the designated meal and drink only water until the end of the study. The breakfast on the day will be the prescribed meal and consumed approximately 1.5~2.5 hours before the scheduled meeting time. The food subject to the study will be consumed by the morning.

9.2.13. Restrictions on Research Subjects during the Study Period

Research institutions, etc. shall instruct research subjects to observe the following items during the study period (from the acquisition of consent to the end of the Phase 2 study).

9.2.13.1. Prohibited items

< During the study period >

1) Overseas travel

< During the on-site examination >

1) Prohibit consumption of alcoholic beverages throughout the day on the day before the examination.
2) Prohibit all food and drink after 21:00 on the day before the examination (Except for water and hot water.).
3) Prohibit all food and drink from the time of awakening until the end of the examination on the day of the examination (Excluding regular meals, water, and hot water).

9.2.13.2. Precautions

< Meals, Drinks, and Favorite Items >

1) Refrain from making major lifestyle changes (Eating too little or too much, starting a new exercise routine or stopping a routine, drastically changing bedtimes, or traveling for more than a week)
2) Refrain from smoking
3) Continue to take supplements and health foods that have been taken continuously before the date of obtaining consent
4) Refrain from using supplements and health foods that may affect the study
   *Examples of supplements and health foods that may affect the study
      MCT and coconut oil (including foods containing high amounts of them)
5) Do not significantly change the frequency of consumption of foods that are known to contain high amounts of medium-chain fatty acids
   *Examples of foods that are known to contain high amounts of medium-chain fatty acids
      Dairy products
6) Supplements and health foods taken should be recorded in the background survey (inspection date).

<Hospital visits, pharmaceuticals, etc.>

1) If the patient visits a medical institution, promptly contact the principal investigator or [28.7. Consultation Desk] (Excluding regular visits to hospitals and health checkups).
2) Refrain from donating blood
3) Ingested drugs (including OTC drugs) should be recorded in the background survey (inspection

day).

Other

1) If for any reason you are unable to consume the food subject to the study or come to the hospital on the inspection day, promptly contact the investigator or the [28.7. Consultation Desk].

10. Preparation of case report form or data list

The investigator should prepare a data list of the results of all research subjects. The completed list should be managed with the date of preparation entered and processed so that it cannot be corrected or changed thereafter. In the unlikely event that a correction is required after completion, the relevant part of the electronic file should be corrected and identified from the original file. The corrected part, reason for correction, version number, and date of preparation should be recorded. The electronic file should be managed with processing so that it cannot be corrected or changed thereafter.

The data list should be consistent with the source documents, and it should be observed that names, etc. that could identify individuals should not be included.

11. Determination of subjects for analysis

After fixing the case report form or data list, discontinuations/dropouts, intake records of the food subject to the study, violations of restrictions during the study period (Prohibited items and precautions), etc. should be confirmed, and the investigator should determine subjects for analysis according to [13.1 Analysis set]. At this time, a record stating the list of subjects for analysis, the reason for exclusion from the analysis, the date of judgment, etc. should be prepared.

12. Key open

The key open should be performed after completing the case report form or data list and determining the subjects for analysis. The allocation manager should disclose the allocation table and identification table.

13. Statistical analysis

13.1. Analysis set

The efficacy analysis set includes study subjects who do not meet the following exclusion criteria. The safety analysis population includes all research subjects who ingested the research food at least once. However, excluding those who withdrew consent and refused to use the test results collected until then.

1. Those who were found to have violated eligibility when selecting research subjects

2. Those who were judged to be inappropriate by the investigator, such as those who conspicuously undermined the reliability of the test results, such as non-compliance with restrictions
3. Those who were judged to be inappropriate as analysis subjects by the investigator for other reasonable reasons

13.2. Evaluation and Analysis

13.2.1. Evaluation and analysis of primary and secondary endpoints

For the blood metabolites and metabolic markers of the primary and secondary endpoints, descriptive statistics (Number of subjects, mean, standard deviation, standard error, maximum, median, minimum) will be calculated for the measured values, changes between pre-intake and post-intake blood sampling points (Δ value), AUC of Δ value (ΔAUC), Cmax, Cmax of Δ value (ΔCmax), and Tmax, and comparisons will be made between the test food and the control food. In addition, the measured values will be compared between pre-intake and post-intake blood sampling points.

For the abdominal symptom questionnaire (graded) of the secondary endpoint, descriptive statistics (Number of subjects, mean, standard deviation, standard error, maximum, median, minimum) will be calculated for pre-intake and post-intake blood sampling points, and maximum values in each study period, and comparisons will be made between the test food and the control food. The measured values will be compared between pre-ingestion and post-ingestion blood sampling points.

As a secondary analysis, the test food, control food, and reference food will be compared in the same manner as above.

13.2.2. Safety assessment and analysis

The symptoms, severity, and frequency of adverse events and adverse drug reactions will be tabulated and evaluated. Details will be specified in a separate statistical analysis plan.

14. Adverse events

14.1. Definition of adverse events

Adverse events are all undesirable or unintentional injuries or diseases or signs thereof (including abnormal laboratory test values) that occur in research subjects, regardless of whether they have a causal relationship to the conducted research. In this study, adverse events are those that newly occur from the day before intake of the food subject to the research to the day after the Day 2 examination.

However, if the symptoms or findings have not worsened since the start of the research, or if the physician involved in the research has made a medical judgment that the occurrence of the event is expected based on the condition of the research subject before the start of the research, the event will not be treated as an adverse event.

14.2. Definition of a serious adverse event

An adverse event is defined as a serious adverse event if it:

1) results in death
2) is life threatening
3) requires hospitalization or prolongation of hospitalization for treatment
4) results in permanent or significant disability or dysfunction
5) results in birth defects in offspring

14.3. Collection and recording of adverse events

In the event of an adverse event, appropriate measures should be taken as necessary, and the following items should be investigated, assessed, and recorded:

1) Symptoms and findings of adverse events

For adverse events, describe symptoms or findings. If the same adverse event occurs more than once, in principle, it should be treated as a separate event. However, if it should be treated as one consecutive event based on the judgment of the investigator, the reason for the judgment should be recorded.

2) Date of occurrence of adverse event

If the date of occurrence is unknown, confirm the date of occurrence confirmation and record that it is the date of confirmation. For symptoms that were observed before the start of the study, the date of occurrence should be the date on which they worsened.

3) Degree

Mild: Symptoms that can be easily tolerated and do not interfere with daily life

Moderate: Some interference with daily life

Severe: Inability to function in daily life

4) Continuation or discontinuation of the study

Continuation

Discontinuation

Study completion

5) Treatment

None

Yes If yes, investigate and record the details

6) Causal relationship with the food under study

For all adverse events, determine the causal relationship with the food under study according to the following five categories. If judged as "not related," "probably not related," or "not evaluable," record the rationale.

Not related : When there appears to be little or no apparent temporal relationship, or other

factors (Complications, concomitant drugs, concomitant foods, etc.) are clearly possible.

Probably not relevant: When there is no apparent temporal relationship, or other factors (Complications, concomitant drugs, concomitant foods, etc.) are highly possible.

Probably relevant: When there is an apparent temporal relationship, and the involvement of other factors (Complications, concomitant drugs, concomitant foods, etc.) can be largely excluded.

Relevant : When there is an apparent temporal relationship, and the involvement of other factors (Complications, concomitant drugs, concomitant foods, etc.) can be clearly excluded.

Not evaluable : When causality with the food under study cannot be determined due to lack of evaluation materials.

7) Outcome, date of outcome

Judgment and recording on the following six levels. When signs or symptoms observed before the start of the study worsen, "recovery" is defined as the time when the investigator judges that the condition at the start of the study has recovered. In the case of recovered, recovered with sequelae, or death, record the outcome date. In the case of improved, not recovered, or unknown, record the date on which the outcome was confirmed as the outcome date.

Recovered

Improved

Not recovered

Death

Recovered with sequelae

Unknown

8) Comments

Describe the details of the treatment, the grounds for determining causality, and the reasons for failure to confirm recovery or remission.

15. Ensuring the safety of research subjects

15.1. Basic matters

During the study period, the investigator should ascertain the condition of the research subjects as much as possible and collect research-related safety information.

When the investigator, etc. becomes aware of the occurrence of an adverse event, the investigator should take appropriate measures to ensure the safety of the research subjects. The investigator should conduct follow-up investigations as much as possible until the safety of the research subjects is

confirmed, such as when the adverse event disappears or the condition before the start of the study is restored or improved. If the event is judged to be a serious adverse event, take appropriate measures according to the [15.2. Response to Serious Adverse Events]

15.2. Response in the event of a serious adverse event

In the event of a serious adverse event, regardless of its causal relationship to the research food, immediately discontinue the research of the research subject and take the following measures.

< Principal investigator >

1) Immediately report to the research representative and the director of the research institution.
2) Additional information should be collected as appropriate and provided to the research representative and the director of the research institution.
3) If a serious adverse event that occurs cannot be predicted and a direct causal relationship cannot be ruled out, the director of the research institution should be notified, and the status and results of the response should be promptly reported to the Minister of Health, Labour and Welfare and published.

< Research representative >

1) After hearing the opinion of the ethics review committee on whether or not it is appropriate to continue the research, the director of the responsible institution should be notified to that effect, and necessary measures should be taken.
2) Information on the occurrence of the adverse event should be shared with the research director of each research institution and all researchers involved in the study.

16. Burden and expected risks and benefits for research subjects

16.1. Burden and expected risks for research subjects

16.1.1. Events caused by ingestion of the study food

The control and test foods contain MCT. MCT may cause abdominal symptoms such as heartburn, bloating, abdominal pain, constipation, and diarrhea after ingestion.

16.1.2. Physical distress and risk of infection associated with blood sampling

Blood sampling is performed by physicians, nurses, or laboratory technicians at the research institution. In this study, blood sampling is performed by puncture, which may cause symptoms that can occur in common blood sampling tests, such as bleeding spots, hematoma, electrical shock, numbness, pain, and discomfort.

16.1.3. Mental distress associated with measurements and investigations

Patients may experience mental distress due to fasting, time constraints, or the contents of various measurements and investigations.

16.2. Projected benefits

The results of blood tests conducted in this study will enable study subjects to understand their own health status. In addition, if the usefulness of MCT coated with milk protein can be confirmed as a result of this study, it will be possible to develop foods containing MCT that are easier to consume, and it is expected that this will contribute to the promotion of people's health.

16.3. Comprehensive Assessment and Measures to Minimize Burden and Risk

< Measures to Minimize Burden and Risk >

This study will cause the burdens and risks described in [16.1. Burden and anticipated risks to research subjects]. As a method to minimize the burdens and risks, it has been confirmed that no adverse events were observed in a previous study in which 6 g of MCT, the same dose as the target food used in this study, was administered continuously for 3 months [5,6]. Even if they occur, abdominal symptoms are transient, and accidents should be prevented by taking appropriate measures based on the judgment of the investigator. Accidents should be prevented by conducting blood sampling by qualified medical personnel. Regarding events caused by measurement and investigation, the subjects should be informed before the start of the study that only those items necessary for conducting the study will be examined and investigated, and that all examinations will not be forced and can be withdrawn at any time. As fasting time will be long, refreshments will be provided to the subjects at the examination site after the examination to ensure their health and safety when returning home.

< Overall evaluation >

When considering the burdens and risks that occur to the subjects mentioned above, methods to minimize them, and the expected benefits, the expected benefits and effects are higher than the burdens and risks, and the significance of conducting the study is considered.

17. Withdrawal and dropout of individual subjects

17.1. Definition

Withdrawal means "Discontinuation of the study at the discretion of the investigator before completion of the study."

Dropout means "If the research is terminated due to the convenience or will of the research subject before the completion of the research."

17.2. Discontinuation criteria for individual research subjects

The investigator shall discontinue the research of the research subject if any of the following events occurs:

1) If the research subject withdraws consent after the commencement of the study
2) If the result of the PCR test performed at the pre-stay examination is positive
3) If a serious adverse event occurs
4) If an adverse event occurs and the investigator determines that the study should be discontinued
5) If it is discovered after the start of the study that the subject is not eligible for the study
6) If the subject does not visit the study site on the day of the visit inspection
7) If it is discovered that the subject has serious or persistent non-compliance with the study protocol
8) In other cases where the investigator deems it necessary to discontinue

17.3. Discontinuation procedure for individual research subjects

1) Discontinuation should be explained to the research subjects. Physical condition and intake status of the research foods should be investigated by interview or interview.
2) The reason for discontinuation, the contents of observation and examination until discontinuation, and the date of discontinuation should be recorded.

17.4. Response to dropout

When the investigator confirms the intention of the research subject to withdraw from the research, the investigator should immediately discontinue the research of the research subject and record the date of withdrawal. However, if the investigator determines that it is necessary to confirm the safety of the research subject in consideration of the safety of the research subject, the investigator should explain this to the research subject and conduct the necessary investigation after obtaining their understanding.

When the research subject indicates the intention to withdraw consent, the investigator should confirm the handling of the data obtained by the time of consent withdrawal with the research subject and respond accordingly.

18. Discontinuation of the entire research

The research representative shall discontinue the research if he/she discovers serious or continued non-compliance with the research protocol, contract, etc., by the research conducting organization or the research operating organization, etc., and determines that this has hindered the proper conduct of the research. Serious non-compliance refers to violations that threaten the human rights, safety, and welfare of the research subjects and the reliability of the research. Continued non-compliance,

regardless of its seriousness, refers to repeated non-compliance without taking appropriate measures despite the research representative's request for improvement.

If the research director determines that the research must be discontinued or interrupted for unavoidable ethical or medical reasons, such as ensuring the safety of the research subjects, the research director shall discontinue or interrupt the research and report this to the research director and the research director of each research institution.

If a reason arises that forces the research director to discontinue the research as a whole, the research director shall consult with the research governing body to determine the discontinuation of the research as a whole and the post-discontinuation measures.

The research director shall promptly notify the director of each research institution and the ethical review committee in writing of the discontinuation of the research as a whole and the reason therefor. Upon receiving the notification, the research director shall provide the necessary information to each research subject and discontinue the research in accordance with the [17.3. Withdrawal procedures for individual study subjects].

If any request is made by the ethical review committee regarding post-discontinuation measures, the research director shall consult with each research institution and respond accordingly.

19. Adherence to, revision of, deviation from, or modification of the research protocol

19.1. Agreement and adherence to the research protocol

The research representative shall consult with the research director of each research institution and prepare a research protocol. The research representative shall submit the research protocol agreed with the research director of each research institution to the ethical review committee for approval.

All researchers, etc. involved in this research shall comply with the research protocol approved by the ethical review committee.

19.2. Revision of the research protocol

The research representative shall revise the research protocol when deemed necessary in conformity with the following cases, etc. It should be noted that when revising, the revision is repeated.

1) When he/she becomes aware of matters related to the quality and safety of the food to be used in the research, or other important information for the proper conduct of the research.
2) When requested by the research director of each research institution.
3) When the plan is changed based on the opinion of the ethics review committee.

When the plan is revised, follow [19.1 Agreement and Compliance with the Research Protocol].

19.3. Deviation from the research protocol

In unavoidable circumstances, such as to avoid immediate danger to research subjects, the investigator may deviate from the research protocol without prior agreement with each research institution and prior approval from the ethical review committee. In such cases, the investigator should report the details and reasons for the deviation to the research representative and the head of the research institution as soon as possible.

If the investigator deviates from the research protocol, he/she should record all deviating actions and report them to the research representative. The research representative should report them to the head of the research institution. The head of the research institution should promptly seek the opinion of the ethical review committee and take appropriate measures for any deviation that he/she judges to have a significant impact on the conduct or results of the research or to significantly increase the risk to the research subjects.

If the deviation causes continuous changes in the research protocol, the research representative should promptly revise the research protocol and submit it for review by the ethical review committee.

20. Ethical conduct of research

20.1. Ethical review and permission or report from the head of the research institution

20.1.1. Compliance with ethical guidelines

This research will be conducted in compliance with the "Ethical Principles Based on the Declaration of Helsinki" and the "Ethical Guidelines for Life Science and Medical Research Involving Human Subjects."

20.1.2. Review by the ethical review committee and permission from the director of the research institution

This research will be conducted upon submission of the following documents to the ethical review committee, review from the viewpoint of ethical and scientific appropriateness, and approval from the director of each research institution.

・Research protocol

・Outline of the food subject to the research

・Written explanation of consent

・Written consent

・Written withdrawal of consent

・Written monitoring procedure

20.1.3. Report to the head of the research institution

If the research representative obtains the following information, he/she shall report it to the director of each research institution without delay.

・Information that may affect the continuation of the research

・Occurrence of serious adverse events

・Inappropriate conduct (Non-Conformity in the Guidelines for Life and Medical Sciences and Deviations from the Research Protocol)

・Management of the storage of human samples and information

・Completion of the study

・Progress of the study if the implementation period is one year or more

20.1.4. Report to the ethics review committee

The research representative or the director of the responsible institution will seek the opinion of the ethics review committee if the following information becomes available:

・Information that may affect the continuation of the study

・Serious adverse events

・Non-compliance with the Guidelines for Biological and Medical Sciences

・Deviations that are judged to materially affect the conduct or results of the study or to significantly increase the risk to the study subjects

The research representative shall report the following to the ethical review committee:

・Completion of the study

・Progress of the study if the implementation period is one year or more

20.2. Consent for research participation

In selecting research subjects, the appropriateness of asking them to participate in the study should be carefully considered from the viewpoint of protecting human rights and based on the inclusion and exclusion criteria in this research protocol, taking into account the health status, age, ability to judge the explanatory consent document, dependency with the research representative, and whether they will participate in research involving other people.

20.2.1. Contents to be included in the explanatory consent document

The research physician should prepare an explanatory consent document containing the items specified as "matters to be explained to the research subjects, etc. when obtaining informed consent" in the "Ethical Guidelines for Life Science and Medical Research Involving Humans."

20.2.2. Timing and Method of Obtaining Consent

The principal investigator or research collaborator designated by the principal investigator shall provide sufficient explanation to the prospective participants based on the Consent Explanation Document, confirm that the prospective participants have fully understood the content, and then obtain written consent of their own free will.

The research subjects shall sign the consent form and enter the date of consent. The principal investigator or research collaborator designated by the principal investigator who provided the explanation shall sign or affix his/her name and seal and enter the date. The original consent form shall

be retained by the research institution and a copy shall be retained by the research subjects.

20.2.3. Points of Attention Regarding Obtaining Consent

The principal investigator or research collaborator designated by the principal investigator who provided the explanation shall not coerce or unduly influence the research subjects to participate or continue to participate in the research.

When providing verbal information regarding the Consent Explanatory Document and the Consent Form, use words and phrases that are understandable to the research subjects, and do not use expressions that would cause the research subjects to waive their rights or that would absolve or suspect the legal responsibility of the research institution. In providing the explanation, the principal investigator or research collaborator designated by the principal investigator shall provide the research subjects with an opportunity to ask questions and sufficient time to make a decision about participating in the research.

20.2.4. Revising the Consent Explanatory Document and the Consent Form

If important information related to obtaining consent is obtained, the research physician will promptly revise the Consent Explanation Document, and after prior review by the ethical review committee, the research physician will promptly provide the research subject with another explanation using the revised Consent Explanation Document and obtain consent for continued research participation. However, this will not apply if the director of the research institution judges that it is not necessary to obtain re-consent after receiving the opinion of the ethical review committee.

20.3. Matters Related to the Protection of Human Rights of Research Subjects

20.3.1. Basic Matters

Persons involved in this research shall give due consideration to the protection of personal information of research subjects when handling consent forms, source documents, etc., and providing and disclosing data. In the preparation of case reports, etc., and the publication of research papers, etc., measures shall be taken to protect personal information of research subjects by preventing identification of research subjects, such as using IDs unique to the research subjects.

20.3.2. Management of Identification and Correspondence Table

Cooperating institutions shall give consideration to the protection of privacy of research subjects, assign IDs to each research subject so that individuals cannot be identified at a glance, and store data, samples, etc. of research subjects obtained through this research. The correspondence table of personal information and IDs shall be appropriately managed by cooperating institutions in lockable areas. When storing as electronic information, passwords shall be set in electronic files, and then stored in external electronic media and stored in lockable areas.

20.4. Compensation and Insurance for Health Damage

If any health damage occurs to a research subject as a result of his/her participation in this research, the research physician shall promptly treat the damage and take other necessary measures, and shall report to each research institution. If any health damage due to this research occurs and liability for compensation or compensation arises, it shall be borne by the research institution responsible for the research, except in cases where the responsibility is attributable to the research institution or the research operating institution. If any responsibility cannot be determined, each research institution shall discuss in good faith and resolve the matter. However, this shall not apply if there is no causal relationship with this research, or if it can be determined that the health damage occurred due to the research subject's own negligence or intention. In order to fulfill the liability for compensation or compensation, the research institution responsible for the research shall consult with each research institution and take necessary measures such as insurance.

In addition, if any problem that is not described in this research protocol occurs during the research period or after the completion of the research, each research institution shall consult with each other and take appropriate measures.

20.5. Matters related to incidental findings obtained in this research

In the event that findings that have a significant impact on the health of research subjects are incidentally found, we will contact the research subjects upon their request.

21. Storage and disposal of samples and information obtained in this research

21.1. Storage and Disposal of Samples Obtained in the Study

Measures should be taken to protect the personal information of research subjects by preventing them from being identified using IDs, etc.

After blood collection, plasma should be separated and submitted to the responsible institution without delay. The responsible institution should keep the blood under lock until measurement and dispose of it without delay after measurement.

Other blood should be submitted to the clinical laboratory measurement institution without delay. After measurement, the blood should be disposed of appropriately in accordance with the regulations of the clinical laboratory measurement institution.

21.2. Storage and disposal of information and records

The research institution shall retain records of operations related to this research, such as original documents such as consent forms, investigation and measurement records, and the research protocol until five years have elapsed from the completion or discontinuation of this research.

The research operating institution shall retain documents to be retained by the research operating institution until five years have elapsed from the completion or discontinuation of this research.

The research responsible institution shall retain documents to be retained by the research responsible institution (such as ID data received from the research institution) until five years have elapsed from the completion or discontinuation of this research.

When disposing of stored materials, each research institution should use an appropriate method (e.g., shredding) to prevent leakage of personal information.

21.3. Creation of records concerning the transfer of samples and information

When the research institution provides samples and information obtained in this study to other research institutions, records should be created and stored at both the source and the destination each time samples and information are transferred.

22. Secondary use of information obtained in this study

The information obtained in this study may be used for additional studies on the absorbability of MCT, but the details have not yet been decided.If secondary use is to be conducted, a new protocol will be created and an ethical review will be conducted.

23. Direct access of source documents, etc.

Upon request from the research representative or the ethical review committee, the research institution and the research operating organization shall accept surveys and provide direct access to source documents, etc. (Consent form, abdominal symptom questionnaire, data used for evaluation, etc.), records of ethical review, records of permission from the head of the research institution, etc. However, from the viewpoint of protecting personal information, sufficient consideration shall be given to direct access to source documents, etc. containing personal information.

24. Monitoring

The monitoring will be conducted after the "Monitoring Procedures" are submitted to the ethical review committee and approved.

25. Conflicts of interest

This study will be funded by contract research funds from the responsible institution. The operating institution will pay for this study to each relevant institution based on a contract with the research director.

Although the funding for this study will be borne by the responsible institution, the responsible institution and the operating institution can only advise on the study, and the independence of the

physicians and the research will be ensured. Therefore, there is no conflict of interest that would affect the results of this study or the interpretation of the results among all persons involved in this study and their families, or among all contractors and the operating institution.

26. Registration of the outline, progress, and results of this study

The responsible institution will register the outline of the study in UMIN-CTR prior to the implementation of the study. Any changes in the progress of the study or the study plan will be updated accordingly. When the study is completed, the results will be registered without delay.

27. References

1. Courchesne-Loyer, A.; Lowry C.M.; St-Pierre V.; Vandenberghe C.; Fortier M.; Castellano C.A.; Wagner J.R.. Cunnane S.C. Emulsification Increases the Acute Ketogenic Effect and Bioavailability of Medium-Chain Triglycerides in Humans: Protein, Carbohydrate, and Fat Metabolism. *Current developments in nutrition* **2017**, 1, e000851.
2. Pi-Sunyer, F.X.; Hashim S.A.. Van Itallie T.B. Insulin and ketone responses to ingestion of medium and long-chain triglycerides in man. *Diabetes* **1969**, 18, 96-100.
3. Owen, O.E.; Morgan A.P.; Kemp H.G.; Sullivan J.M.; Herrera M.G.. Cahill G.F., Jr. Brain metabolism during fasting. *J. Clin. Invest.* **1967**, 46, 1589-1595.
4. Henderson, S.T.; Vogel J.L.; Barr L.J.; Garvin F.; Jones J.J.. Costantini L.C. Study of the ketogenic agent AC-1202 in mild to moderate Alzheimer's disease: a randomized, double-blind, placebo-controlled, multicenter trial. *Nutr. Metab. (Lond.)* **2009,** 6, 31.
5. Abe, S.; Ezaki O.. Suzuki M. Medium-Chain Triglycerides in Combination with Leucine and Vitamin D Benefit Cognition in Frail Elderly Adults: A Randomized Controlled Trial. *J. Nutr. Sci. Vitaminol. (Tokyo)* **2017**, 63, 133-140.
6. Abe, S.; Ezaki O. Suzuki M. Medium-Chain Triglycerides in Combination with Leucine and Vitamin D Increase Muscle Strength and Function in Frail Elderly Adults in a Randomized Controlled Trial. *J. Nutr.* **2016,** 146, 1017-1026.
7. Naohisa Nosaka; Kuki; Yoshie Suzuki; Michio Kasai; Aoyama Toshiaki; Kazuo Kondo; Motoko Taguchi; Sato Mitsuru. Ichiro Kono. Effects of Triacylglycerol Intake Containing Medium-Chain Fatty Acids on Muscle Thickness and Blood Composition in Male Wrestlers. *Japanese Journal of Clinical Nutrition* **2011,** 33, 12-21.
8. Ashitani, J.; Matsumoto N.. Nakazato M. Effect of octanoic acid-rich formula on plasma ghrelin levels in cachectic patients with chronic respiratory disease. *Nutr. J.* **2009,** 8, 25.
9. Miki, K.; Kitada S.; Miki M.; Hui S.P.; Shrestha R.; Yoshimura K.; Tsujino K.; Kagawa H.; Oshitani Y.; Kida H.; Maekura R.. Kangawa K. A phase II, open-label clinical trial on the

combination therapy with medium-chain triglycerides and ghrelin in patients with chronic obstructive pulmonary disease. *J. Physiol. Sci.* **2019**, 69, 969-979.

10. Kawai, K.; Nakashima M.; Kojima M.; Yamashita S.; Takakura S.; Shimizu M.; Kubo C.. Sudo N. Ghrelin activation and neuropeptide Y elevation in response to medium chain triglyceride administration in anorexia nervosa patients. *Clinical nutrition ESPEN* **2017**, 17, 100-104.
11. Maher, T.; Deleuse M.; Thondre S.; Shafat A.. Clegg M.E. A comparison of the satiating properties of medium-chain triglycerides and conjugated linoleic acid in participants with healthy weight and overweight or obesity. *Eur. J. Nutr.* **2021**, 60, 203-215.
