# Supplementary material for: Ketogenic effects of milk protein–coated medium-chain triglycerides in healthy men: A randomized, double-blind, crossover study
Source: PLoS One. 2026 Aug 10;21(8):e0355475. doi: 10.1371/journal.pone.0355475 (PMC13456251; doi:10.1371/journal.pone.0355475)
Supplement: Supplementary file 3 [file pone.0355475.s003.docx]

**S3 Fig. Microscopic images of coated MCT.** Lipids were stained with BODIPY 493/503 (green), and proteins were stained with Nile Blue A (red). Both were photographed with a confocal microscope. MCT, medium-chain triglycerides.
