# Supplementary material for: Ketogenic effects of milk protein–coated medium-chain triglycerides in healthy men: A randomized, double-blind, crossover study
Source: PLoS One. 2026 Aug 10;21(8):e0355475. doi: 10.1371/journal.pone.0355475 (PMC13456251; doi:10.1371/journal.pone.0355475)
Supplement: Supplementary file 4 [file pone.0355475.s004.docx]

**S4 Fig. Time courses of (A) active ghrelin and (B) desacyl ghrelin.** Values are presented as means ± standard deviation. **P* < 0.05, untreated MCT vs. coated MCT, Wilcoxon signed-rank test; ^#^*P* < 0.05, vs. LCT. Wilcoxon signed-rank test (Bonferroni correction). MCT, medium-chain triglycerides; LCT, long-chain triglycerides.
