# Supplementary material for: Ketogenic effects of milk protein–coated medium-chain triglycerides in healthy men: A randomized, double-blind, crossover study
Source: PLoS One. 2026 Aug 10;21(8):e0355475. doi: 10.1371/journal.pone.0355475 (PMC13456251; doi:10.1371/journal.pone.0355475)
Supplement: Supplementary file 6 [file pone.0355475.s006.docx]

**S6 Table. Participant background (Details)**

|  |  | U to C sequence (*n* = 6) | | | C to U sequence (*n* = 5) | | | *P*-value |
| --- | --- | --- | --- | --- | --- | --- | --- | --- |
| Systolic blood pressure | mmHg | 117.2 | ± | 15.4 | 117.2 | ± | 12.3 | 0.748 |
| Diastolic blood pressure | mmHg | 65.7 | ± | 6.1 | 66.3 | ± | 11.9 | 0.423 |
| White blood cell | /μL | 5800 | ± | 1554 | 5617 | ± | 1658 | 0.748 |
| Red blood cell | 104/μL | 481 | ± | 40 | 484 | ± | 36 | 1.000 |
| Hemoglobin | g/dL | 14.9 | ± | 1.2 | 14.5 | ± | 1.3 | 0.630 |
| Hematocrit | % | 45.9 | ± | 4.0 | 44.5 | ± | 3.5 | 0.689 |
| Platelet | 104/μL | 29.8 | ± | 5.1 | 25.6 | ± | 5.5 | 0.230 |
| Total protein | g/dL | 6.8 | ± | 0.7 | 7.1 | ± | 0.3 | 0.334 |
| Albumin | g/dL | 4.2 | ± | 0.4 | 4.3 | ± | 0.3 | 0.746 |
| AST | U/L | 19.8 | ± | 4.6 | 18.0 | ± | 2.0 | 0.809 |
| ALT | U/L | 23.3 | ± | 7.3 | 22.5 | ± | 6.9 | 1.000 |
| γ-GTP | U/L | 24.7 | ± | 8.0 | 29.2 | ± | 17.0 | 0.687 |
| LDH | U/L | 167.2 | ± | 37.5 | 150.2 | ± | 18.3 | 0.378 |
| T-Bil | mg/dL | 0.72 | ± | 0.21 | 0.93 | ± | 0.28 | 0.224 |
| ALP | U/L | 73.8 | ± | 15.1 | 71.5 | ± | 25.7 | 0.873 |
| Urea nitrogen | mg/dL | 10.0 | ± | 2.2 | 12.1 | ± | 2.2 | 0.128 |
| Creatinine | mg/dL | 0.78 | ± | 0.15 | 0.87 | ± | 0.14 | 0.230 |
| Uric acid | mg/dL | 5.8 | ± | 0.5 | 6.3 | ± | 0.6 | 0.168 |
| Na^+^ | mEq/L | 141.5 | ± | 2.1 | 141.2 | ± | 1.2 | 0.934 |
| Cl^-^ | mEq/L | 104.0 | ± | 2.2 | 104.0 | ± | 1.1 | 0.870 |
| K^+^ | mEq/L | 4.0 | ± | 0.3 | 4.0 | ± | 0.2 | 1.000 |
| Ca^2+^ | mg/dL | 9.3 | ± | 0.5 | 9.4 | ± | 0.2 | 0.809 |
| Triglycerides | mg/dL | 132.3 | ± | 51.8 | 94.2 | ± | 17.6 | 0.093 |
| Total cholesterol | mg/dL | 175.5 | ± | 34.9 | 191.8 | ± | 38.1 | 0.689 |
| HDL-cholesterol | mg/dL | 54.0 | ± | 19.9 | 57.2 | ± | 12.4 | 0.936 |
| LDL-cholesterol | mg/dL | 99.7 | ± | 28.0 | 115.3 | ± | 32.3 | 0.298 |
| HbA1c | % | 5.3 | ± | 0.3 | 5.2 | ± | 0.2 | 0.373 |

Values are presented as mean ± standard deviation. Comparisons between sequences were made using the Wilcoxon rank sum test. U, untreated medium-chain triglycerides (MCT); C, coated MCT; AST, aspartate aminotransferase; ALT, alanine aminotransferase; γ-GTP, amma-glutamyl transpeptidase; LDH, lactate dehydrogenase; T-Bil, total bilirubin; ALP, alkaline phosphatase; HDL, high-density lipoprotein; LDL, low-density lipoprotein; HbA1c, glycated hemoglobin.
